# Networking for a successful career in clinical hematology: a strategic approach

**DOI:** 10.46989/001c.141400

**Published:** 2025-07-24

**Authors:** Mohamad Mohty, Rahul Banerjee

**Affiliations:** 1 Sorbonne Université; Centre de Recherche Saint-Antoine, INSERM UMRs 938; Service d’Hématologie Clinique et de Thérapie Cellulaire, Hôpital Saint Antoine, AP-HP, Paris, France; 2 Fred Hutchinson Cancer Center, Seattle, WA, USA

**Keywords:** networking, career development, mentorship

## Abstract

Networking is fundamental to career development in clinical hematology, providing avenues for knowledge exchange, collaborations, and professional growth. This manuscript examines specific strategies for networking within this specialized field, detailing effective platforms, strategies, overcoming challenges, and illustrating real-world success stories. References to key studies and expert opinions underscore the importance of building a robust professional network through in-person and online activities. An extensive review of literature highlights how networking contributes to scientific collaboration, mentorship, career opportunities, and the dissemination of cutting-edge hematological research.

## Introduction

Networking is a critical element in the career trajectory of clinical hematologists. The field demands continuous education, collaborative research, and innovative patient care approaches. Establishing a broad professional network can likely facilitate access to cutting-edge research, clinical trials, and multidisciplinary collaborations, all essential for career progression. Additionally, networking favors intellectual growth by exposing hematologists to diverse perspectives and novel approaches in diagnosis, treatment, and management of hematologic disorders. It bridges the gap between early-career professionals and seasoned more advanced experts, ensuring the transfer of knowledge and skills essential for high-quality patient care.

## Benefits of networking in clinical hematology

Networking is closely linked to mentorship, providing early-career hematologists with guidance from experienced professionals.^1,2^ Mentorship relationships often extend beyond academic advice to include career planning, skill development, and emotional support.^3^ Collaborative research is enhanced through joint grants and multi-center studies. These collaborations can lead to high-impact publications, increased funding opportunities, and novel therapeutic advancements.^4^ Networking also facilitates access to funding sources, industry partnerships, and participation in clinical trials, which are pivotal for translational research. Knowledge exchange through conferences and publications ensures up-to-date clinical practice, helping hematologists adopt best practices and innovative treatments. A strong network also offers emotional, professional and leadership support, enhancing job satisfaction and reducing burnout.^5,6^ Networking creates a community of practice where hematologists can share challenges, celebrate successes, and seek advice.

Major conferences like the American Society of Hematology (ASH) Annual Meeting (www.hematology.org), the European Hematology Association (EHA) Congress (www.ehaweb.org) or the International Academy for Clinical Hematology (IACH) annual meeting and diverse activities (www.iach.org), provide unparalleled networking opportunities. These events host thousands of hematologists, offering a platform to meet peers, discuss research, and form collaborations. Such professional associations offer member-exclusive resources, webinars, and mentorship programs. Membership in these associations provides access to journals, clinical guidelines, and funding opportunities (e.g. https://iach.org/mobility-grant/). On the other hand, online platforms facilitate broader and global connections, allowing hematologists to join specialized groups, follow thought leaders, and share their research. For example, ResearchGate supports academic collaborations by enabling researchers to share their work (www.researchgate.net), seek feedback, and collaborate on projects. Additionally, hospital seminars, university events, and local workshops provide accessible and practical networking opportunities, fostering relationships within the local medical community.

## Building a professional network in person

Active involvement in professional societies and presenting at conferences are important strategies to build networks. Regularly attending industry events and participating in panel discussions or advisory boards (if permitted) can increase visibility as well. Maintaining regular contact with mentors and peers is crucial. Sending follow-up emails, sharing updates, and scheduling periodic check-ins help sustain relationships. Furthermore, leveraging alumni networks, participating in volunteer opportunities within professional organizations, and seeking leadership roles in committees can significantly enhance one’s professional standing.

Successful networking requires a proactive approach. Initiating conversations, reaching out to colleagues, and attending industry events are essential strategies. Following up with personalized messages after meetings shows genuine interest and helps solidify connections. Offering value to peers, such as sharing relevant articles, providing assistance with research, or introducing them to other professionals, enhances networking efforts. Consistency in attending events and engaging online builds lasting relationships. Developing strong interpersonal skills, such as active listening, empathy, and effective communication, is crucial for successful networking. Additionally, preparing elevator pitches, keeping business cards handy, and setting specific networking goals for each event attended can significantly improve networking efficiency.

At regional and global conferences, networking involves thorough preparation, active engagement in sessions, and participation in social events. Researching attendees, scheduling meetings, and planning one’s schedule enhances networking success. Active participation in sessions through questions, discussions, and presentations increases visibility. Social events like dinners, coffee breaks, and receptions offer informal settings for building connections. Timely follow-ups after conferences with personalized messages and continued engagement help solidify relationships formed during the event. Additionally, spontaneously volunteering as a session chair, presenting posters, and joining conference committees can provide additional networking opportunities and demonstrate leadership. Many conferences have networking events designed specifically for trainees and junior faculty, for example Young European Society for Blood and Marrow Transplantation (EBMT) events in Europe and ASH-a-Palooza at ASH meetings in the United States. These types of sessions are highly recommended given their structured opportunities to meet and network with potential mentors.

Cooperative groups and research societies are another specific avenue for networking that are very valuable to junior faculty. Examples in Europe include l’Intergroupe Francophone du Myélome (IFM) in France and el Programa Español de Tratamiento en Hematología (PETHEMA) in Spain. In the United States, cooperative groups include Alliance, SWOG (Southwestern Oncology Group), and ECOG / ACRIN (Eastern Cooperative Oncology Group / American College of Radiology Imaging Network). In the setting of transplantation and cellular therapies, analogous groups include the EBMT and the Center for International Blood and Marrow Transplantation Research (CIBMTR). These research groups encourage junior faculty to collaborate on research projects, serve as study champions, or pursue secondary analyses of published data. As examples within multiple myeloma, many recent publications from the CIBMTR Plasma Cell Disorders Working Group have included junior faculty as first authors.^7,8^ Similarly, junior faculty have been first authors of recent secondary analyses from published SWOG and BMT CTN trials.^9,10^

## Building a professional network online

Establishing a strong online presence by sharing research, writing blog posts, and participating in discussions enhances visibility. Building a network requires time and effort but yields long-term benefits, including career advancement, collaborative opportunities, and personal growth.^11^ Social media platforms like LinkedIn and X (formerly Twitter) (or similar platforms) are valuable tools for networking. LinkedIn allows hematologists to join groups, follow key opinion leaders, and share their research. X offers real-time updates from conferences, discussions on emerging topics, and direct interactions with peers. Specific hashtags related to a given disease/field or a specific congress (e.g. #Hematology, #ASH24, #EHA24 and #IACH24) facilitate finding relevant content and discussions. Social media not only broadens networking horizons but also ensures continuous learning and engagement with the global hematology community.^12^ Creating content, such as short videos explaining research findings or blog posts discussing clinical experiences, can attract a wider audience and foster meaningful connections.

As a word of caution in an increasingly polarized world, two of the challenges with online networking in hematology involve staying focused and staying (mostly) positive. We encourage junior faculty to post, like, comment, and ask questions about clinical and research topics relevant to them. With regard to the universe of other topics that may interest any given physician – sports, film, politics, and more – in most cases, it may be easier to consolidate this content onto other social media platforms or via a separate account. Commonly used social media platforms for our field to discuss hematologic issues include Twitter/X, BlueSky, and LinkedIn; this of course does not restrict the use of other platforms for personal use. Secondly, one of the advantages of online networking is the democratization of scientific conferences^12^ Junior faculty and senior leaders in the field can communicate easily on public platforms that others (including other hematologists and of course patients) can see. Hematologists whose social media posts largely focus on dismissing studies and the physicians who run such studies, for instance, are viewed negatively and lose their ability to actually effect the changes for which they are advocating. Online networking in hematology is most successful when one is earnest and focuses one’s attention on learning and on advancing the field, not on criticizing others.

## Overcoming networking challenges

Challenges such as introversion, time constraints, and fear of rejection can hinder networking efforts. Introverted professionals can start with small, manageable interactions such as online networking or one-on-one meetings. Allocating time for networking amidst busy schedules is essential, and tools like calendar reminders can help. Overcoming the fear of rejection involves understanding that most professionals value connections and are open to building relationships. Developing confidence through practice and preparation can further ease networking anxiety.^13^ Additionally, finding networking partners or peers with similar challenges can create a support system and make networking less daunting. The final outcome can be very rewarding, and there are many examples of younger hematologists who leveraged social media to secure participation into multi-center research partnership, leading to a high-impact publication and career advancement. Finding a career-changing mentor during a networking session, resulting in collaborative projects and leadership roles is another frequently cited example. These cases highlight the profound impact of networking on career success and underscore the importance of strategic relationship-building. Another classical well-known scenario involves junior researchers who used X (formerly Twitter) to join global discussions on hematology, leading to invitations for national and international speaking engagements and collaborations. Finally, some networking apps are being developed (e.g. Shapr and Meetup) to help professionals find like-minded individuals and industry events. Books such as “*Never Eat Alone*” by Keith Ferrazzi provide timeless networking advice. Online sessions by the IACH offer practical tips and strategies for effective networking. Utilizing these tools enhances networking efforts and ensures continuous professional growth. Additionally, subscribing to newsletters from professional societies, participating in online forums, and using digital tools like virtual business cards can further facilitate effective networking.

## Conclusion

Like in many other fields, networking is useful (if not mandatory) for career advancement in clinical hematology. Building and maintaining professional connections fosters continuous learning, collaborative opportunities, and career growth. Similar to clinical training, investing some time and effort in networking is an investment in a successful future, enabling hematologists to stay at the forefront of their field, contribute to innovative research, and ultimately provide optimal patient care. A well-established network not only benefits individual careers but also advances the field of hematology as a whole by promoting shared knowledge and collaborative innovation.

### STATEMENTS AND DECLARATIONS

The authors declare no competing financial interests in relation with this work.

### ETHICAL APPROVAL

Not applicable.

### CONSENT TO PARTICIPATE/INFORMED CONSENT

Not applicable.

### CONSENT FOR PUBLICATION

Not applicable.
